# Prevention of exposure to and spread of COVID-19 using air purifiers: challenges and concerns

**DOI:** 10.4178/epih.e2020027

**Published:** 2020-04-17

**Authors:** Seunghon Ham

**Affiliations:** Department of Occupational and Environmental Medicine, Gil Medical Center, Gachon University College of Medicine, Incheon, Korea

**Keywords:** COVID-19, Air purifier, Occupational disease, Infection cluster, Call centers, Occupational health

## Abstract

Coronavirus disease 2019 (COVID-19) is now a pandemic. The Korean government has declared a red alert, which is the highest level of the national infectious disease alert system, and the World Health Organization has similarly declared its highest-level pandemic alert (phase 6). The spread of COVID-19 is an unprecedented worldwide public health problem that governments and individuals must work to overcome. Recently, an infection cluster arose in a call center in Seoul. To support call center companies, the Korean Ministry of Employment and Labor has proposed covering the costs of installing partitions and air purifiers, providing hand sanitizers, and supplying masks to prevent droplet and aerosol infections. Air purifiers are expected to be installed on the floor with the exhaust outlets at a higher level, such as the level of the desks or breathing zones of workers. When a worker coughs or releases droplets near a colleague’s respiratory system, the droplets may spread throughout the call center via air flow from air purifier. In this fashion, a single infected person can give rise to an infection cluster. Attempts to prevent infection must not lead to new infections, and the installation of air purifiers may cause new problems. Therefore, using air purifiers to control the spread of COVID-19 should be approached with caution.

The first case of coronavirus disease 2019 (COVID-19) in Korea was confirmed on January 20, 2020. On February 23, 2020, the Korean government issued a red alert (the highest level of the infectious-disease alert system according to the Korea Centers for Disease Control and Prevention), and on March 11, 2020, the World Health Organization issued a pandemic alert of the highest level (phase 6). COVID-19 has become a global issue, and international cooperation is currently crucial [1-4].

Since the original primary focus of interventions against COVID-19 was the prevention of community infection, the development of policies to prevent infection from occupational exposure has been neglected. A recent outbreak in a Seoul call center exemplifies occupation-related disease, as it was caused by occupational exposure to a biological hazard. Due to the nature of the job, workers at the call center interacted extensively in a high-density space, increasing the probability of infection. In addition, the work environment likely contributed to the infection cluster in that taking breaks during business hours at a call center is rare, which made it more difficult for the workers to seek treatment.

As of March 16, 2020, Korea’s Ministry of Employment and Labor proposed to pay 20 million won to more than 1,100 small and medium-sized call center companies with fewer than 50 full-time workers to cover the costs of installing partitions and air purifiers and providing hand sanitizers and masks for the prevention of droplet and aerosol infection. This seemed to be an appropriate measure to protect the health of workers in crowded call centers.

However, using air purifiers without considering the heating, ventilation, and air conditioning (HVAC) system of the facility may expose call center workers to new risks. The use of air purifiers is not an appropriate approach to control COVID-19 at call centers for the following reasons.

First, air purifiers use a dilution ventilation method, which reduces the concentration of harmful substances in the air, while diluting particulate matter (such as fine dust) or gaseous substances (such as volatile organic compounds). Diluted ventilation is widely used due to its low toxicity, low emission rate, and high effectiveness for gaseous substances, and it is used when the substances are produced uniformly over time or when the installation of a local exhaust ventilation system is difficult. The virus that causes COVID-19 is a highly contagious, small, and a biological hazard that is still not fully understood. Therefore, air purifiers employing dilution ventilation are unsuitable. Methods of controlling biological hazards such as COVID-19 are, from most to least effective, elimination, substitution, isolation, engineering control (ventilation), administrative measures, and the use of personal protective equipment (PPE). To prevent community infection, the most feasible option is to recommend the use of PPE, even though this is less effective than other methods; however, call centers can be adequately managed through the proper application of isolation and ventilation.

The second challenge is posed by the air dispersion methods of such air purifiers. Most air purifiers function by directing pollutant-containing air downwards to capture it, passing the air through a filter, and discharging the purified air back into the surroundings. Since the purified air must be discharged at a greater distance from the purifier than its intake, the wind speed is stronger at the outlet than at the inlet. Therefore, since relatively strong air flow is present at the outlet, the pressure difference causes the air to rise and disperse into the surroundings at a location farther away or positioned higher than the inlet.

A pilot experiment showed that the flow of water mist into an air purifier inlet depended on the height of the source (Figure 1). Air purifiers are relatively complex because of the structure of the air flow involved. Figure 1A and 1B illustrates the flow of water mist into an air purifier inlet. Of note, when the height of the water vapor source was increased, the water vapor did not flow into the inlet of the air purifier. Instead, the air flow in the exhaust zone was disturbed due to the strength of the outflow (Figure 1C and 1D). The distance of the water vapor from the air purifier inlet made the efficiency lower than expected. The exhaust of the air purifier had a relatively high wind speed, with a maximum of approximately 10 m/s. In light of these findings, it may be possible to distribute air without filtering out the virus that causes COVID-19. Considering that most air purifiers are installed at the floor level, the installation of air purifiers to limit the spread of COVID-19 seems to have more drawbacks than advantages. This experiment was a pilot study and therefore had several limitations, including the use of water mist to mimic and visualize the droplets, the lack of evaluation of a variety of air purifiers, and the need to replicate the experiment. Nevertheless, given that COVID-19 is an urgent social and public health issue that requires debate, I believe that this pilot experiment was sufficiently meaningful to start a discussion.

Since the government only covers installation costs for air purifiers and does not provide detailed research-based guidelines, most air purifiers will likely be installed on the floor, not at the desk level. If a worker coughs or releases droplets in close proximity to the face of a co-worker, the droplets could disperse throughout the space of the call center via the air flow. In this fashion, one infected person can give rise to an infection cluster.

Therefore, to prevent COVID-19 spread in call centers, control at the source is most effective since anyone may be infected. Potential control measures include the modification of existing partitions into a booth configuration or the installation of simple booths with preinstalled local exhaust ventilation systems. This partitioning can be compared to the sudden drop in the suction power of a vacuum cleaner with an increase in distance. The distance between inlet and the pollutant is the one of most important aspects of ventilation theory. It is necessary to implement a design that does not inconvenience workers, and considering the social impact of asymptomatic infected individuals, this effort should be undertaken by the government, employers, and workers.

In addition, various factors warrant further research, such as filter effectiveness and efficiency, air purifier sterilization capacity, and the transmission of the virus through HVAC systems. Other measures to prevent COVID-19 infection are appropriate, such as installing partitions, providing hand sanitizers, and supplying mask by Ministry of Employment and Labor in Korea; however, the installation of air purifiers may not be suitable and should be approached with caution, as measures designed to prevent infection should not lead to new infections.

## Ethics statement

This paper is a perspective so it did not need ethical consideration.

## Figures and Tables

**Figure 1.**
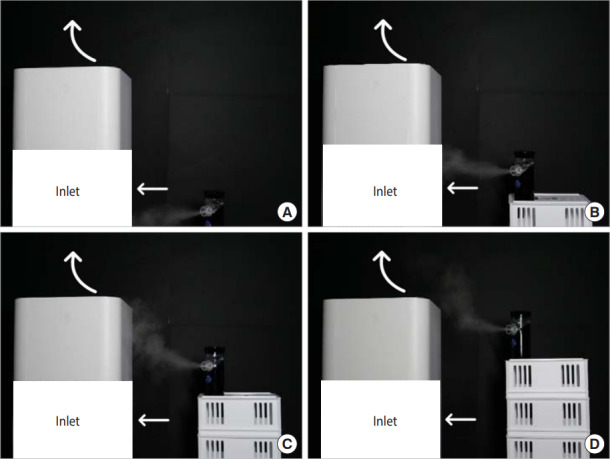
Pilot experiment result of visualized air flow in terms of height of source (A) floor level, (B) 8 cm, (C) 16 cm, and (D) 24 cm from floor level.
